# dbPepNeo2.0: A Database for Human Tumor Neoantigen Peptides From Mass Spectrometry and TCR Recognition

**DOI:** 10.3389/fimmu.2022.855976

**Published:** 2022-04-13

**Authors:** Manman Lu, Linfeng Xu, Xingxing Jian, Xiaoxiu Tan, Jingjing Zhao, Zhenhao Liu, Yu Zhang, Chunyu Liu, Lanming Chen, Yong Lin, Lu Xie

**Affiliations:** ^1^College of Food Science and Technology, Shanghai Ocean University, Shanghai, China; ^2^Shanghai-Ministry of Science and Technology (MOST) Key Laboratory of Health and Disease Genomics, Institute for Genome and Bioinformatics, Shanghai Institute for Biomedical and Pharmaceutical Technologies, Shanghai, China; ^3^School of Health Science and Engineering, University of Shanghai for Science and Technology, Shanghai, China; ^4^Bioinformatics Center, National Clinical Research Centre for Geriatric Disorders, Department of Geriatrics, Xiangya Hospital, Central South University, Changsha, China; ^5^Department of Bioinformatics and Biostatistics, Shanghai Jiao Tong University, Shanghai, China

**Keywords:** neoantigen, mass spectrometry, experimental validation, TCR, deep learning

## Abstract

Neoantigens are widely reported to induce T-cell response and lead to tumor regression, indicating a promising potential to immunotherapy. Previously, we constructed an open-access database, i.e., dbPepNeo, providing a systematic resource for human tumor neoantigens to storage and query. In order to expand data volume and application scope, we updated dbPepNeo to version 2.0 (http://www.biostatistics.online/dbPepNeo2). Here, we provide about 801 high-confidence (HC) neoantigens (increased by 170%) and 842,289 low-confidence (LC) HLA immunopeptidomes (increased by 107%). Notably, 55 class II HC neoantigens and 630 neoantigen-reactive T-cell receptor-β (TCRβ) sequences were firstly included. Besides, two new analytical tools are developed, DeepCNN-Ineo and BLASTdb. DeepCNN-Ineo predicts the immunogenicity of class I neoantigens, and BLASTdb performs local alignments to look for sequence similarities in dbPepNeo2.0. Meanwhile, the web features and interface have been greatly improved and enhanced.

## Introduction

The complex process by which the immune system eliminates cancer cells is regulated by several factors; one of the most important events is the production of neoantigens (1, 2). Different mutated variants of numerous mutations lead to the production of new protein sequences, which were deemed as the primary sources of neoantigens (3, 4), such as single-nucleotide variants (SNV) and insertions or deletions (INDEL). The non-coding regions of the genome is another source of neoantigens, such as long non-coding RNAs (2, 4, 5). These foreign proteins are naturally processed by antigen-presenting cells (APC) cells and presented by human leukocyte antigens (HLAs) and then truly induce an efficient response of *CD8^+^ T* or *CD4^+^ T* lymphocytes (6–8). Theoretically, tumor neoantigens are promising cancer immunotherapy targets due to being not subject to central tolerance and less likely trigger autoimmune toxicity (7, 9–11).

At present, there are two primary approaches for utilizing tumor neoantigen in clinical practices: one is to expand and reinforce natural or modified T cells *ex vivo* and then reinject them back into the patient to kill cancer cells (12–15). Another is to design and develop personalized vaccines (16, 17). In 2021, Hu et al. showed that neoantigen peptide vaccines can induce persistent memory T-cell responses and expand the range of tumor-specific cytotoxicity (18). Meanwhile, further investigations found that a combination therapy of personalized neoantigen vaccines and checkpoint blockade monoclonal antibodies could also enhance T-cell responses in patients (17). Moreover, neoantigens play significant roles in immune escape and immunoediting (19, 20).

Mass spectrometry (MS)-based workflows applied on clinical cancer cell lines and tumor samples produce rich peptidomics data sets and hence the straightforward identifications of MHC-bound peptides (21). Integrating with next-generation sequencing, this approach has reported dozens of neoantigens deriving from immunopeptidomic analysis (22–25). In addition, fusion is an important class of somatic mutations formed by chromosome structural variation (SV) (11), which can serve as an ideal source of neoantigens for creating an open reading frame (ORF) (11, 26, 27). Wei et al. showed that neoantigens derived from fusion genes tend to have notably higher immunogenic potential than common single-nucleotide variation and indel-based candidate neoantigens, making them more viable as clinical cancer vaccines (11, 27, 28). Laumont et al. identified 40 specific neoantigens based on the proteogenomic approach, which mainly derived from allegedly non-coding regions (29). The diversity of discovering the source of tumor neoantigens implies that T cells can be directed against a variety of genomic aberrations in cancer (30). Therefore, there is great interest in integrating all immunogenic peptide resources for cancer immunotherapy.

First released in 2019 by our group, dbPepNeo was a database of human tumor neoantigens either immunogenically tested or mass spectroscopically validated, which contained high-confidence (HC) neoantigens, medium-confidence (MC) neoantigens, and low-confidence (LC) HLA-binding peptidomes. With the increasing need for breadth and precision of neoantigen data, we updated dbPepNeo (31) to version 2.0 here. dbPepNeo2.0 substantially added HC and LC data, greatly expanded the data volume and application scope, and significantly modernized the dbPepNeo user interface. In addition to expansion of existing data types, dbPepNeo2.0 also added novel data types such as fusion neoantigen peptides, HLA class II neoantigens, and non-coding region neoantigens. Also, neoantigen-reactive T-cell receptor-β (TCRβ) sequences were firstly included. Furthermore, two new neoantigen analytical tools were incorporated into dbPepNeo2.0 to provide selection of the immunogenic neoantigens. DeepCNN-Ineo predicts the immunogenicity of class I neoantigens, and BLASTdb performs local alignments to look for sequence similarities in dbPepNeo2.0. All of these additions and enhancements are intended to support the development of neoantigen-based cancer vaccines and facilitate research in clinical cancer immunotherapy.

## Materials and Methods

### Data Acquisition and Classification

We updated dbPepNeo2.0 mainly by compiling the new data from peer-reviewed immunology literatures, and the existing public databases (e.g., Cancer Antigenic Peptide Database, Immune Epitope Database) (32, 33). Different combinations of keywords were used to perform the search in PubMed (neoantigen, tumor, neoepitope, epitope, peptidomes, peptidomics), mass spectrometry-related words, and restricted species (human cancer). Indexed papers have a limited time range (between January 2010 and June 2021).

**Figure 1:**
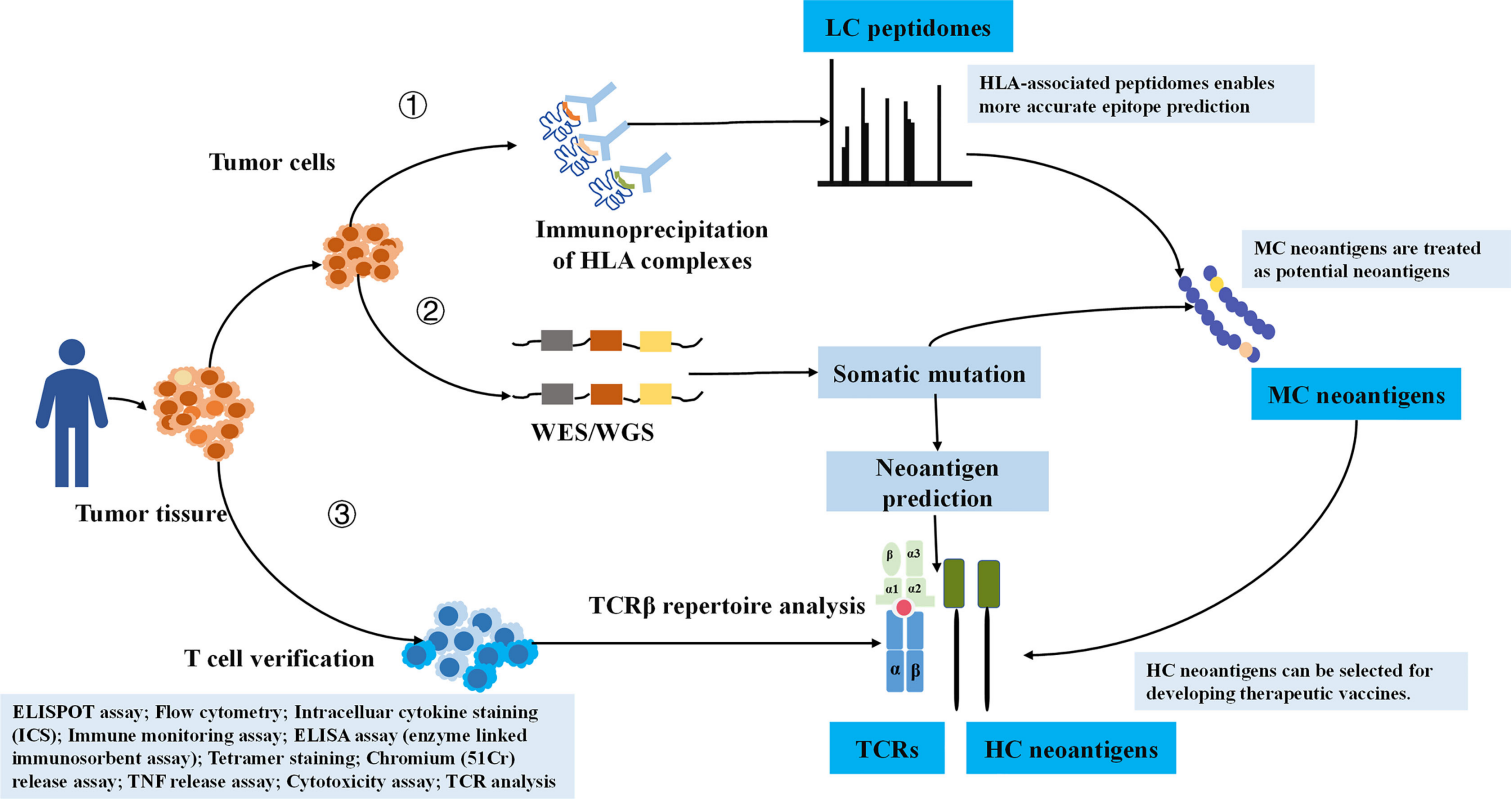
The illustration of HC, MC neoantigens, and LC immunopeptidomes based on validation approaches. LC, low confidence; MC, medium confidence; HC, high confidence.

In order to ensure the reliability of the data, we manually checked each retrieved literature (31). Simply, the curated peptides were classified into three degrees of confidence according to verification methods (**Figure 1****)**. We defined LC immunopeptidomes as raw peptides bound by HLA molecules and identified by MS; MC neoantigens were peptides with somatic mutations and verified by MS and whole exome sequencing (WES)/whole genome sequencing (WGS); and HC neoantigens were those immunogenic peptides validated by specific TCR recognition experiments (31). Neoantigen immunogenicity manifests the interaction of neoantigens HLA with TCR, which concretely performs as T-cell proliferation, T-cell activation, TNF release, IFN-γ release, granzyme B release, IL-2 release, and IL-10 release (**Figure 1**).

### Data Annotation

All screened papers were further read and manually curated, and each neoantigen peptide was provided with additional annotation. Original data details were integrated including neoantigen peptides, wild peptide sequence, peptide length, mutant position, HLA allele, gene name, cancer or tumor type, methods of verification, PubMed ID, and the reference links. Furthermore, we used NetMHCpan (v4.1) and NetMHCIIpan (v4.0) to provide mutated peptide affinity (half-maximal inhibitory concentration) IC50 (nM), %rank, and binding level, respectively (34). For class I peptides, peptides with %rank less than 0.5 will be considered as strong binding (%rank  ≤ 0.5, SB), 0.5  <  %rank  ≤  2 will be considered as weak binding (WB), and %rank > 2 will be considered as no binding (NB). For class II peptides, the top 2% of the predicted peptides will be identified as strong binder (%rank ≤2), then peptides will be considered as weak binder if the %rank is above 2 but below the specified 10 (2% < rank ≤ 10%) (35).

To further study the immune response of T cells to neoantigens, we collected validated T-cell receptor-β (TCRβ) complementarity-determining region 3 (CDR3) sequences specific for tumor neoantigens (class I or II) and firstly constructed TCRβ-CDR3 sequence libraries. Eventually, we unified retrieved information and each entry was provided with cancer type, gene name, neoantigen sequence, CDR3 sequence, variable region of TCR (TRBV), diversity region of TCR (TRBD), and joining region of TCR (TRBJ) (36), as well as the reference links, if available in literatures.

### New Tools DeepCNN-Ineo and BLASTdb Were Added in dbPepNeo2.0

Affinity HLA–peptide interactions have been reported to be positively correlated with immune responses (37). Thus, a web-based tool, DeepCNN-Ineo (deep-learning model for predicting immunogenicity of neoantigens based on convolutional neural network), was initially developed based on dbPepNeo2.0, which aims at further reducing false positive neoantigen peptides based on processing pipeline prediction and narrowing down of the scope of immunogenicity peptide validation.

The quantitative immunogenic/non-immunogenic neoantigen peptides were collected from dbPepNeo2.0 and PubMed. To restrict the dataset for prediction, manually extracted neoantigen peptides from research articles need to be further processed. 9-mer- and 10-mer-length neoantigen peptides covering 97.5% of the total number of neoantigens (38) obtained from dbPepNeo2.0 were used as training datasets. All HLA molecular typing should have 4-digit alleles; the different HLA alleles with the same neoantigen peptides were considered as different neoantigens. A total of 583 immunogenic neoantigens and 2,200 non-immunogenic neoantigens were retained after removing duplicated data. By comparing different encoding strategies, we finally used the AAindex-encoding strategy to extract amino acid comprehensive physicochemical properties and HLA paratopes (**Supplementary 1**, **Figure S1A**), whose approach was successfully validated in different literatures (39–41). In addition, we considered the binding affinity between HLA–peptide pairs and the potential immunogenicity of peptide-HLA (42). Binding affinity was included into DeepCNN-Ineo; these mass spectrometry data contain not only information about peptide–MHC-binding events (34) but also information about the steps of the biological antigen presentation process. We took the %rank score of binding affinity as a highly reliable reference for neoantigen identification and then the predicted score of the immunogenicity model as a filter. Users can freely choose whether to refer to binding affinity (%rank) or not. Double filtering can increase the reliability of DeepCNN-Ineo prediction of neoantigen immunogenicity.

**Figure 2:**
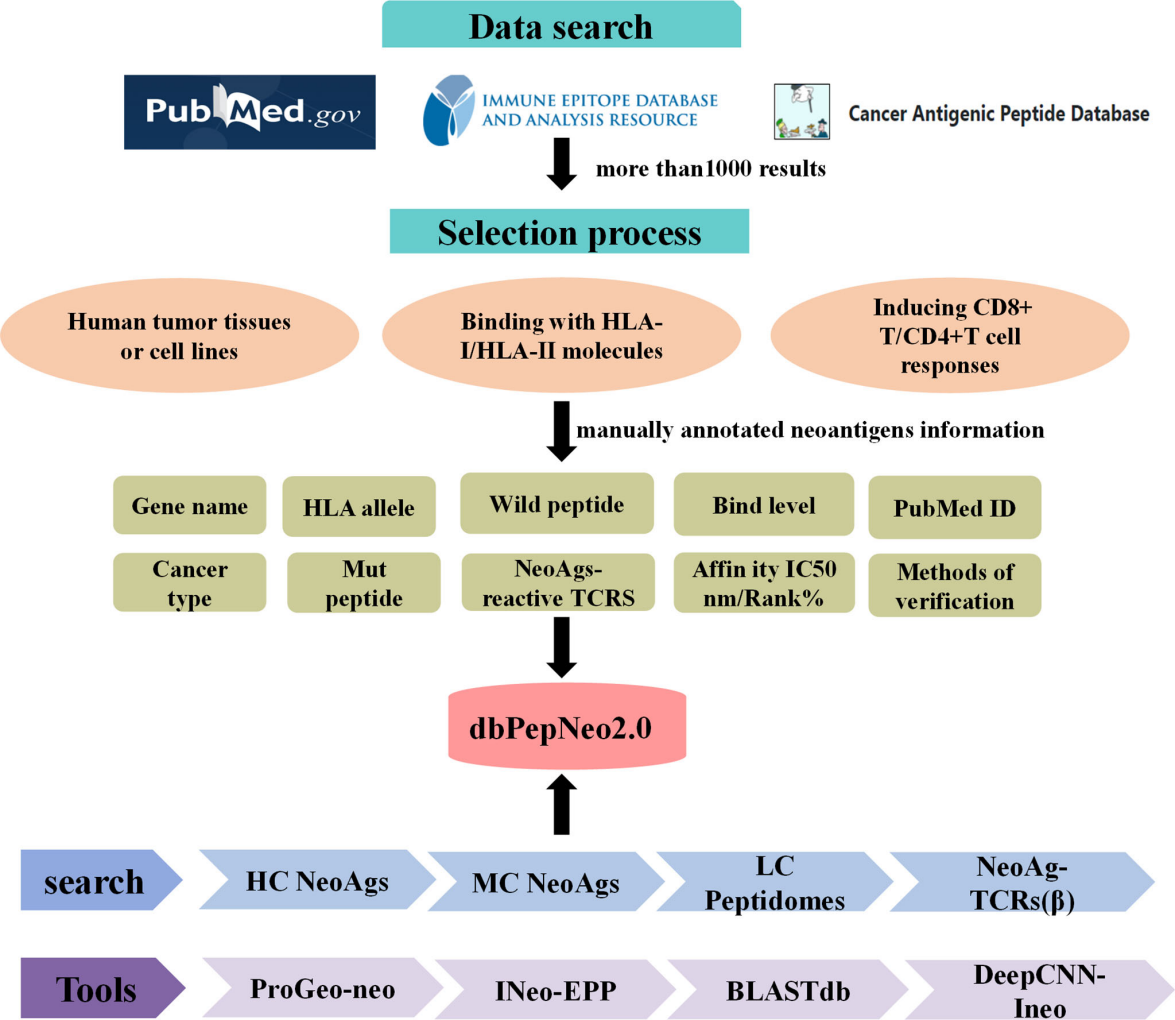
dbPepNeo2.0 content and construction. HC NeoAgs: high-confidence neoantigens; MC NeoAgs: medium-confidence neoantigens; LC Peptidomes: low-confidence immunopeptidomes.

Meanwhile, dbPepNeo2.0 integrated the BLASTdb tool into the database (43). The target sequence database, i.e., reference library, was established with HC or MC neoantigen peptides, while predicted neoantigen peptides were regarded as retrieval sequences. Then, the sequences of homology between predicted neoantigen peptides and the target sequence can be identified by BLASTdb. The structural framework of the dbPepNeo2.0 database is shown in **Figure 2**.

## Results

### Expansion of Existing Data and Addition of New Data to dbPepNeo2.0

**Figure 3:**
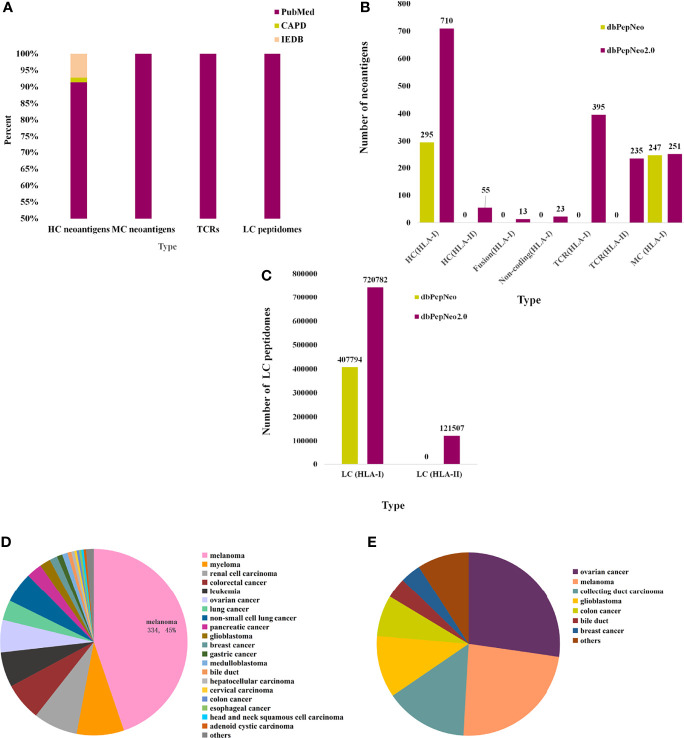
Data summary in dbPepNeo2.0. **(A)** The percentage of neoantigen data collected from different sources in dbPepNeo2.0. **(B)** Number of HC, MC neoantigens, and TCRs in two versions of dbPepNeo. **(C)** Number of LC immunopeptidomes in two versions of dbPepNeo. **(D)** Tumor type of class I HC neoantigens. **(E)** Tumor type of class II high-confidence neoantigens.

dbPepNeo2.0 provides a systematic, quality-controlled catalog of validated neoantigen peptides, TCRs, and HLA peptidomes, which distinguishes from other antigen databases such as IEDB and CAPD; the source of neoantigen data is shown (**Figure 3A**). HC neoantigens are high-confidence neoantigen peptides validated by specific TCR recognition. MC neoantigens are medium-confidence peptides, which involved somatic mutations and are verified by MS and WES/WGS. LC immunopeptidomes are raw peptides bound by HLA molecules and identified by MS. The data changes over these few years are summarized in dbPepNeo2.0 **(** **Figures 3B, C****)**. For this year’s release, dbPepNeo2.0 mainly contained both class I and class II neoantigens and TCRs. For class I peptides, HC neoantigen data types in dbPepNeo2.0 have been greatly increased. HC neoantigens grew from 295 HC neoantigens to 746 HC neoantigens, in which we firstly included 23 neoantigens derived from non-coding regions and 13 neoantigens derived from fusion genes. The number of class I MC neoantigens increased slightly from 247 to 251. dbPepNeo2.0 totally collected 28 immunopeptidomes datasets of human cancer; the number of LC immunopeptidomes grew from 407,794 to 720,782. For the newly added class II peptides, dbPepNeo2.0 firstly collected 55 HC neoantigens and 121,507 LC immunopeptidomes.

Totally, the latest HC neoantigens (classes I and II) involved 30 cancer types and about 45% HC neoantigens were derived from melanoma **(** **Figure 3D****)**. This corresponds to a nearly 1.5-fold increase. Interestingly, we newly included 55 class II HC neoantigens that can induce CD4+ T-cell response **(** **Figures 3B, E****)**. Importantly, each neoantigen peptide provides original detailed details, including cancer type, gene name, HLA allele, wild-type peptide sequence, mutated peptide sequence, mutant position, peptide length, methods of verification, PubMed ID, and the reference links. Also in our database, we can further investigate the specific recognition of HLA–peptide complexes by TCRs. dbPepNeo2.0 firstly added a T-cell receptor-β (TCRβ) library, which encompassed 395 neoantigen TCRβ clonotypes isolated in CD8+ T cells and 235 neoantigen TCRβ clonotypes cloned from CD4+ T cells. Similarly, each entry was provided with cancer type, gene name, neoantigen sequence, CDR3 sequence, TRBV, TRBD, and TRBJ, as well as the reference links, if available in literatures. Overall, both tumor neoantigen coverage and immune response targets were significantly expanded in dbPepNeo2.0.

### Statistics of the Collected HC, MC Neoantigens, and TCRs

**Figure 4:**
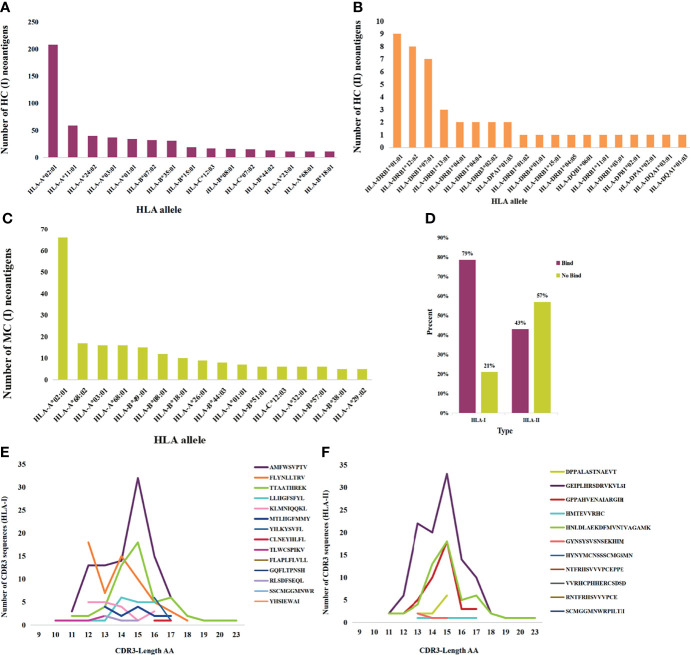
Statistics analysis of the collected HC, MC neoantigens, and TCRs in dbPepNeo2.0. **(A, B)** Top 15 HLA types binding to HC neoantigens. **(C)** Top 15 HLA types binding to MC neoantigens. **(D)** Affinity prediction with NetMHCpan of HC class I and class II neoantigens. **(E)** Length distribution of HLA-I neoantigen-reactive CDR3 sequences. **(F)** Length distribution of HLA-II neoantigen-reactive CDR3 sequences.

Neoantigens that elicit T-cell responses represent the gold standard for developing vaccines. HC neoantigens are immunogenic peptides that were validated by specific TCR recognition. We further analyzed the neoantigens and TCR in dbPepNeo2.0; a total of 71 HLA types in HC neoantigens and MC neoantigens were found. Peptide binding to the HLA*A02:01 molecule accounted for the main proportion. The top 15 alleles with the most frequent HLA binding to class I and class II are shown in **Figures 4A–C**. Meanwhile, %rank was calculated by NetMHCpan (v4.1) and NetMHCIIpan (v4.0) for HLA I and II-restricted neoantigens. The results showed that the class I and class II neoantigens with high affinity to HLA molecules accounted for 79% and 43%, respectively (**Figure 4D**). The prediction performance of class II peptides binding to the MHC software tool needs to be improved. MC neoantigens were defined by MS and WES/WGS and contained somatic mutations, which are treated as potential neoantigens for applying immunotherapy. MS identification of directly eluted cancer-associated HLA peptides defined them as LC immunopeptidomes; these raw peptides are likely presentable to the tumor cell surface, but not guaranteed to elicit a potent T-cell response, until tested empirically. Furthermore, we counted the number and length of neoantigen-reactive CDR3 sequences and found that the majority of CDR3 sequences are composed of 15 amino acids (**Figures 4E, F****)**.

## Interface Enhancements and New Features

**Figure 5:**
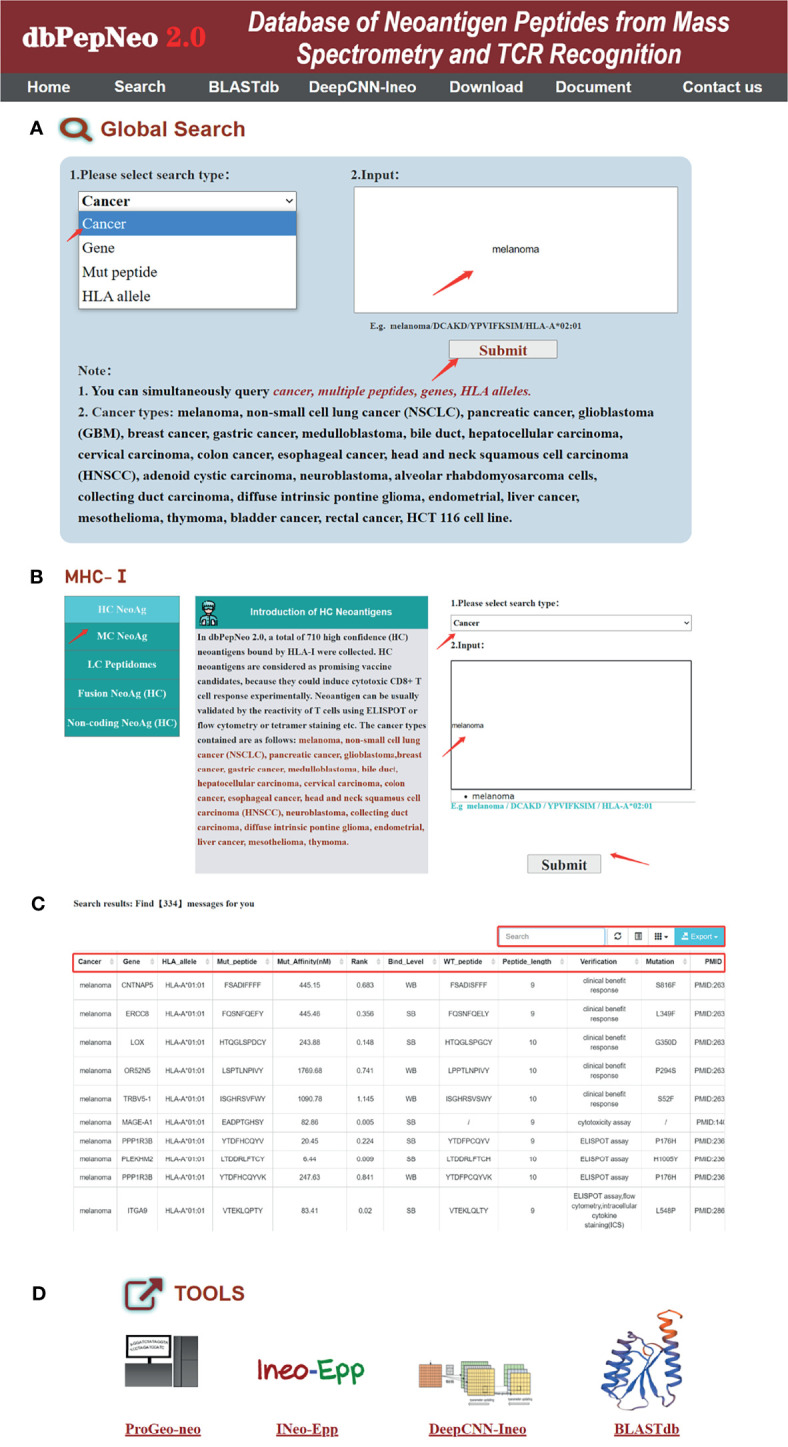
User interface of the dbPepNeo2.0 database; the home page includes seven features. **(A)** Global search is a quick search box; search options include cancer, gene, mut peptide (mutant peptide), and HLA allele. **(B)** The accurate search page for neoantigens; one can choose to search class I and II neoantigens or TCRs. **(C)** Search results for melanoma. **(D)** The four neoantigen prediction and study tools can be utilized on the home page.

To date, the quality of a database is dependent not only on the quality of their content but also on the user-friendliness of their interfaces. In version 2.0 of dbPepNeo, the database’s user interface has been significantly enhanced and expanded. The dbPepNeo2.0 interface has been redesigned; the web interface comprises 7 main pages: (I) Home, (II) Search, (III) BLASTdb, (IV) DeepCNN-Ineo, (V) Download, (VI) Document, and (VII) Contact us. On the Home page, users can quickly find information of tumor-specific neoantigens **(** **Figure 5A****)**. The choices of tumor types, HLA types, mut peptides (neoantigens), and PubMed ID are provided in drop-down menus to simplify the query. Also, users can precisely select the type of data to be retrieved by clicking on the search button, including class I neoantigens, class II neoantigens, and TCRs (**Figure 5B**). Due to the diverse spellings of many cancer types, neoantigen sequences and gene names can be complex or non-intuitive; dbPepNeo2.0 now supports an “intelligent” text search, which automatically provides possible options to supplement the input of incomplete names. Furthermore, the results from database queries have also been enhanced. Some important neoantigen peptides and TCR features are displayed on the result page; users can view complete information *via* the hyperlink on the ID. Detailed information included cancer information, sequence information, verification method, and reference (**Figure 5C**). Then the result page can be further queried by selecting text fields, and the text fields will be highlighted (red font) in the selected result page.

In order to adapt to various needs and preferences of users, the neoantigen analytical tools have been modified for release 2.0 to allow four different types of neoantigen prediction and study tools developed by our group to be used (**Figure 5D**). On the home page, users can quickly utilize analysis tools: ProGeo-neo is a pipeline for proteogenomic neoantigen prediction using MS data (44), and INeo-Epp is a tool for predicting the immunogenicity of epitopes based on the characteristics of antigenic peptides (45). DeepCNN-Ineo and BLASTdb were firstly incorporated into dbPepNeo2.0. In DeepCNN-Ineo, collected data were divided into the following proportions: 60% for the training set, 20% for the validation set, and 20% for the independent test set (46). We used ROC and the normalized confusion matrix to assess the model of predictivity; the independent test set AUC was 0.779 **(** **Supplementary 1**, **Figures S1B, C****)**, and the unnormalized confusion matrix demonstrated that most of the data were correctly classified, which indicated that the model had a good performance. Due to the number of collected neoantigen datasets being not large enough, the construction of this model is only a preliminary attempt in the database. To provide a useful tool for neoantigen selection, we will further optimize our model when the data volume is expanded in the future. Users can choose options to use the DeepCNN-Ineo on the web page. BLASTdb can be used to search sequence similarity. The output format of BLASTdb query results is custom format 6 (43); users can adjust the expected value threshold, word size, gap costs, and matrix to increase the sensitivity.

### Application of dbPepNeo2.0

#### Broad-Spectrum Filtration of Neoantigens Using BLASTdb

Conventional computer prediction processes produce immeasurable candidate neoantigens, which is almost arduous for immunologists and clinicians to eliminate false positive predictive neoantigen peptides by means of experimental validation. Therefore, candidate neoantigens can be further filtered by our database.

**Figure 6:**
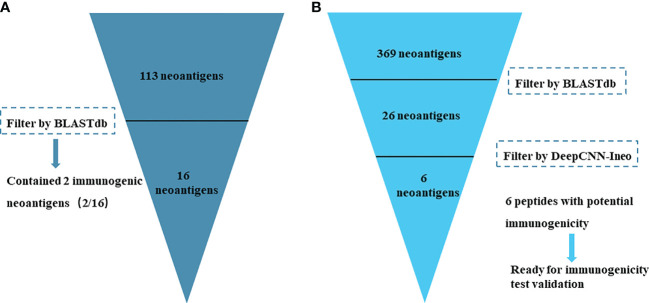
Overview of the results of neoantigen filtering by dbPepNeo2.0 workflow: ProGeo-neo, BLASTdb, and DeepCNN-Ineo. **(A)** Filter results of 113 neoantigens by BLASTdb. **(B)** Filter results of 369 peptides by BLASTdb and DeepCNN-Ineo.

Eight patients with head and neck squamous cell carcinoma (HNSCC) were predicted to produce 113 candidate neoantigens; these 113 candidate neoantigens have been proved using TCR T cells, and two neoantigens have been proved to activate T cells to some extent (47). We obtained the data directly from the published literature without reperforming original data reanalysis. In this case, 113 validated neoantigens were input as query sequences for the BLASTdb tool. All HC neoantigens (801 immunogenic peptides) from dbPepNeo2.0 were used to construct the target sequence library. BLASTdb filter results showed that 16 candidate neoantigen peptides were similar to target sequences (16/113). Particularly, 16 candidate neoantigen peptides also contained 2 neoantigens confirmed by immunoassay experiments (**Figure 6A**). The matching degree of candidate neoantigen and target sequences ranged from 50% to 80% (**Table S1**). The number of filtered neoantigen data was reduced from 113 to 16; this is a much smaller range for potential further immunogenicity validation. Hence, the immunogenic peptides can be further screened by our database, which can greatly reduce the burden of subsequent experimental verification and significantly improve the accuracy of neoantigen prediction.

#### Neoantigen Prediction Using ProGeo-neo, BLASTdb, and DeepCNN-Ineo

ProGeo-neo, dbPepNeo2.0, and DeepCNN-Ineo systematically form a mining pipeline for screening tumor neoantigens. Previously, we used the ProGeo-neo pipeline to predict neoantigens from the Jurkat leukemia cell line; 655 candidate neoantigen peptides were generated (44). As an example, we only took the largest number of 9-mer length candidate neoantigens as the query sequence (369 candidate peptides). Similarly, 801 HC neoantigen peptides (class I and class II) were used as the target sequence library and obtained 26 peptides by BLASTdb filtering (26/369) (**Table S2**). Subsequently, we predicted the immunogenicity of 26 peptides from BLASTdb results using DeepCNN-Ineo (**Figure 6B**). The results showed that 6 mut peptides were identified as immunogenicity-high, and 12 mut peptides were identified as immunogenicity-low; 8 mut peptides were identified as non-immunogenic (**Table S2**). Therefore, in dbPepNeo2.0, we could further identify real neoantigens from p-MHC to reduce the scope for immunogenic peptide validation by using DeepCNN-Ineo (18/369). Compared with the original data, the range of neoantigens used for experimental validation is reduced.

## Discussion and Conclusion

In this work, we updated dbPepNeo to dbPepNeo2.0. We greatly improved and supplemented the database content and optimized the web interface. dbPepNeo2.0 is committed to be an important reference database for tumor neoantigen research, which allows researchers to exploit neoantigens data with mass spectrometry evidence and experimental validation. Moreover, we expect that it can provide guidance on vaccine development in tumor immunotherapy.

dbPepNeo2.0 newly added 13 immunogenic neoantigens derived from gene fusions and 23 neoantigens produced from non-coding regions from all available validated sources. The addition of new data further expanded the boundaries and applicability of neoantigens. In addition, immunogenic neoantigens can elicit *CD8^+^* T- or *CD4*^+^ T-cell responses and produce neoantigen-specific TCR clonotypes. For researchers, validated neoantigen-specific TCR sequences collected in dbPepNeo2.0 can be utilized to explore the specific recognition of neoantigens and accelerate the development of immunotherapy strategies such as TCR-T.

The ultimate purpose of neoantigen presentation by HLA is that they are recognized by TCR and can induce antitumor immune responses (48). We preliminarily constructed a neoantigen immunogenic prediction tool DeepCNN-Ineo based on deep learning, which aims at providing a valuable tool for predicting the immunogenicity of neoantigen peptides. However, it was only a preliminary test on a small scope of fixed datasets from upstream approaches. Owing to the insufficient training data (immunogenic peptides), the performance of our model is not perfect. In addition, HLA*A2 accounts for a very high proportion of the data we can collect. In order to solve the problem of bias in HLA typing, we tried to construct a model with each type separately, but the results were unsatisfactory. The reason for this may be that the characteristics of neoantigens themselves prefer to bind to certain HLA, such as HLA*A02:01, and the amount of data we have is not enough to train a powerful model for all HLA alleles. It is noticeable that TCR sequences of neoantigen-specific recognition are the most important functional feature (39). A model containing TCR information is only suitable for patients with sufficient depth of TCR sequencing. Although high-throughput approaches for single-cell TCR sequencing have been developed, the techniques are still rarely performed in research and clinical settings (39). In addition, the heterogeneity of TCR sequence length and the complexity of cross recognition among TCR sequences both greatly increase the complexity of TCR prediction modeling. Therefore, we have not taken TCRs into account for the time being to predict neoantigen peptide immunogenicity.

Predictive algorithms based on machine learning and artificial intelligence technologies need large training datasets (49), where the data type, quality, and quantity greatly influence the accuracy of prediction (50, 51). Therefore, the continuous accumulation of high-precision data is critical for model construction and optimization (2, 52, 53). Continuous expansion on the biological sources for tumor neoantigen and the necessity for constructing more accurate computational prediction algorithms together stand for the significance and importance of continuous updating of our dbPepNeo, a database for human tumor neoantigen peptides from mass spectrometry and TCR recognition.

## Data Availability Statement

The original contributions presented in the study are included in the article/**Supplementary Material**. Further inquiries can be directed to the corresponding author.

## Author Contributions

LX conceived of the idea and planned and coordinated the entire project. LX, YL, and LC, supervised this study. XJ and XT contributed to the study design. JZ, ZL, YZ, and CL contributed to the data analysis. ML and LFX designed the web interface. LFX wrote the computer program and constructed the database. ML collected and curated data and drafted the manuscript. LX and XJ revised the manuscript. All authors contributed to the article and approved the submitted version.

## Funding

This work was supported by the National Natural Science Foundation of China under Grant [31870829] and Shanghai Municipal Health Commission Collaborative Innovation Cluster Project under Grant [2019CXJQ02].

## Conflict of Interest

The authors declare that the research was conducted in the absence of any commercial or financial relationships that could be construed as a potential conflict of interest.

## Publisher’s Note

All claims expressed in this article are solely those of the authors and do not necessarily represent those of their affiliated organizations, or those of the publisher, the editors and the reviewers. Any product that may be evaluated in this article, or claim that may be made by its manufacturer, is not guaranteed or endorsed by the publisher.
