# Supplementary material for: dbPepNeo2.0: A Database for Human Tumor Neoantigen Peptides From Mass Spectrometry and TCR Recognition
Source: Front Immunol. 2022 Apr 13;13:855976. doi: 10.3389/fimmu.2022.855976 (PMC9043652; doi:10.3389/fimmu.2022.855976)
Supplement: Supplementary file 1 [file DataSheet_1.docx]

**Datasets**

Training and test datasets were analyzed in this study using published experimentally tested neoantigens. The neoantigen data were collected from our dbPepNeo2.0 and PubMed, including 583 immunogenic neoantigens and 2200 non-immunogenic neoantigens. To restrict the dataset for prediction, manually extracted neoantigen peptides from research articles need to be further processed. Neoantigen peptides of length 9-mer and 10-mer were retained for the training model, 9-mer and 10-mer peptides cover 97.5% of all neoantigens counts, which are the dominant length for HLA I-bound peptides (1). All HLA molecular typing should have 4-digit alleles; The different HLA alleles with same neoantigen peptides were considered as different neoantigens.

**Encoding Strategy**

By comparing different encoding strategies, we finally used the AAindex encoding strategy to encode account for amino acid comprehensive physicochemical properties and HLA paratopes (HLA-antigen interacting residues) as a proxy of different HLA alleles, which was successfully validated in the literature (2-4). In addition, we considered the binding affinity between HLA-peptide pairs and the potential immunogenicity of peptide-HLA (5). Binding affinity was included into DeepCNN-Ineo, these mass spectrometry data not only contain information about peptide-MHC binding events (6), but also information about the steps of biological antigen presentation process. We took the %rank score of binding affinity as a highly reliable reference for neoantigen identification, and then the predicted score of the immunogenicity model as a filter. Users can freely choose whether to refer to binding affinity (%rank) or not. Double filtering can increase the reliability of DeepCNN-Ineo prediction of neoantigen immunogenicity.


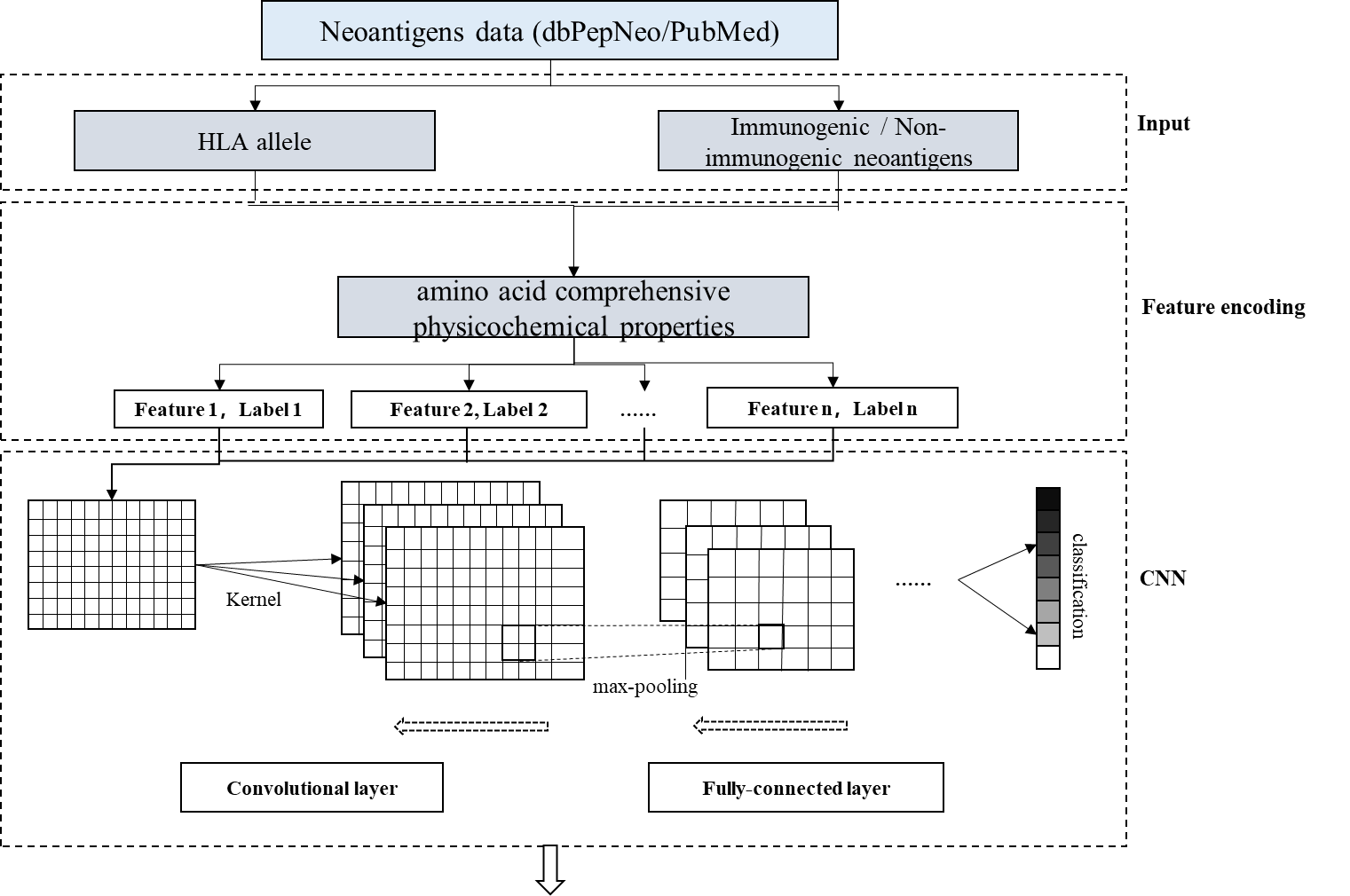


Figure S1 (A) The deep learning model and architecture of convolutional neural network.

**Results**

Peptide and MHC were processed by two consecutive convolutional layers, followed by two dense layers to consider the interactions between peptide and MHC. For the initial feature extraction, we firstly use 32 convolutional filters of stride size 1 to scan along the input feature. The rectified linear unit (ReLU) was chosen as the activation function after the convolutional layer. Next, 64 convolutional filters of stride size 1 were used again. Then, we use the pooling layer to reduce the sample, and both peptide and MHC feature flattened separately to obtain the long-form vectors with flatten layer. After that, two consecutive fully connected layers were operated to generate the output of the convolutional layer.

In DeepCNN-Ineo, collected data were divided into the following proportions: 60% for the training set, 20% for the validation set and 20% for the independent test set. We used ROC and normalized confusion matrix to assess the model of predictivity, and the independent test set AUC was 0.779 **(Fig S1B, S1C)**, and normalized confusion matrix demonstrated that most of the data were correctly classified, which indicated the model had a good performance. DeepCNN-Ineo provides a useful tool for neoantigens recognition and further selection. Users can choose options to use the DeepCNN-Ineo on the web page.


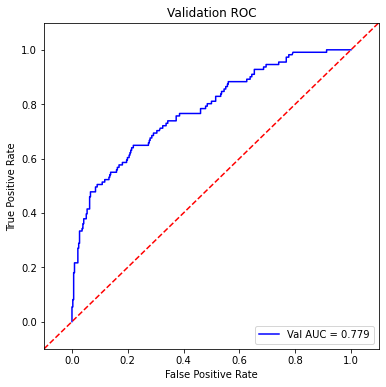


**C**

**B**


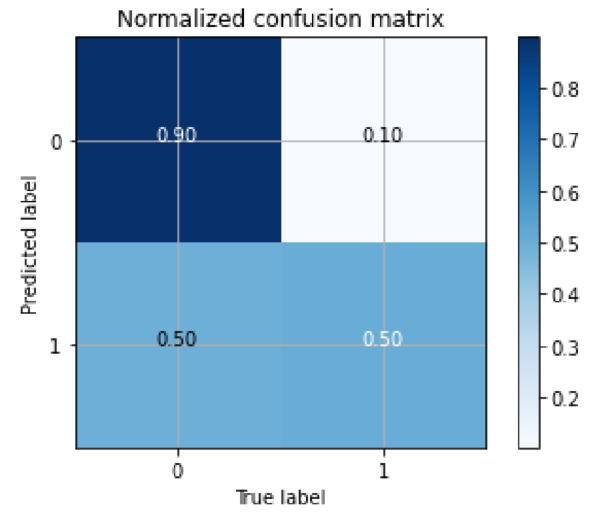


Figure S1 (B) ROC curves of the classifiers for immunogenic prediction in the test dataset. (C) Confusion matrix of the classifiers for immunogenic prediction in the test dataset.

**Reference**

1. Nielsen, M., Lundegaard, C., Blicher, T.*, et al.* (2007) NetMHCpan, a method for quantitative predictions of peptide binding to any HLA-A and -B locus protein of known sequence. *PLoS One*, **2**, e796.

2. Li, G., Iyer, B., Prasath, V.B.S.*, et al.* (2020) DeepImmuno: Deep learning-empowered prediction and generation of immunogenic peptides for T cell immunity. *bioRxiv*.

3. Mei, S., Li, F., Xiang, D.*, et al.* (2021) Anthem: a user customised tool for fast and accurate prediction of binding between peptides and HLA class I molecules. *Brief Bioinform*, **22**.

4. Xu, Z., Luo, M., Lin, W.*, et al.* (2021) DLpTCR: an ensemble deep learning framework for predicting immunogenic peptide recognized by T cell receptor. *Brief Bioinform*, **22**.

5. Wu, J., Wang, W., Zhang, J.*, et al.* (2019) DeepHLApan: A Deep Learning Approach for Neoantigen Prediction Considering Both HLA-Peptide Binding and Immunogenicity. *Front Immunol*, **10**, 2559.

6. Reynisson, B., Alvarez, B., Paul, S.*, et al.* (2020) NetMHCpan-4.1 and NetMHCIIpan-4.0: improved predictions of MHC antigen presentation by concurrent motif deconvolution and integration of MS MHC eluted ligand data. *Nucleic Acids Res*, **48**, W449-w454.
